# A Scoping Review on Malaria Prevention and Control Intervention in Fragile and Conflict-Affected States (FCAS): A Need for Renewed Focus to Enhance International Cooperation

**DOI:** 10.1007/s44197-023-00180-7

**Published:** 2024-01-15

**Authors:** Sanjay Pattanshetty, Viola Savy Dsouza, Anupama Shekharappa, Maheswara Yagantigari, Rohit Raj, Aniruddha Inamdar, Issam Alsamara, Harsh Rajvanshi, Helmut Brand

**Affiliations:** 1 Department of Global Health Governance, Prasanna School of Public Health, Manipal Academy of Higher Education, Manipal, Karnataka India; 2 Centre for Health Diplomacy, Department of Global Health Governance, Prasanna School of Public Health, Manipal Academy of Higher Education, Manipal, Karnataka India; 3 Department of International Health, Faculty of Health Medicine and Life Sciences, Care and Public Health Research Institute-CAPHRI, Maastricht University, Maastricht, The Netherlands; 4 Centre for Regulatory Science, Department of Health Information, Prasanna School of Public Health, Manipal Academy of Higher Education, Manipal, Karnataka India; 5 Saksham Pramaan, Tata Institute of Social Sciences, Mumbai, India; 6 Department of Community Medicine, Manipal Tata Medical College, Manipal Academy of Higher Education, Jamshedpur, India; 7 Asia Pacific Leaders Malaria Alliance, Singapore, Singapore; 8 Department of Health Policy, Prasanna School of Public Health, Manipal Academy of Higher Education, Manipal, Karnataka India

**Keywords:** Malaria, Malaria control interventions, Fragile and conflict-affected states, FCAS, Global Technical Strategy, Scoping review

## Abstract

Malaria is a major public health problem in developing countries. The burden of malaria in fragile and conflict-affected states (FCAS) is increasing year by year. Moreover, the population living in FCAS is often the most vulnerable and at high risk of malaria due to factors, such as deteriorating healthcare system, mass relocations, and reduced resilience to shocks. Therefore, this scoping review aims to map the interventions that are conducted at the FCAS on malaria prevention among the general population. In addition, this review can help policy-makers and international health bodies, providing a comprehensive overview that can lead to more targeted, effective, and context-specific interventions. Databases, such as PubMed, EBSCO-CINAHL, Web of Science, ProQuest, and Cochrane Central Register of Controlled Trials, were searched using specified search terms. A total of 3601 studies were retrieved from the search. After screening, 62 studies were included in the synthesis that met the eligibility criteria. Narrative analysis of the findings was done. The results revealed that in fragile countries, interventions for children below 5 years of age included IPTi, TDA, and ACT. In conflicted countries, interventions for children below 5 years of age included TDA, LLINs, SMC, drug trials, and vaccination. Similar interventions were reported for other age groups and populations. Despite ongoing conflicts, malaria interventions have been maintained in these countries, but a persistent high burden of malaria remains. To achieve the goals of malaria elimination, the results of the review highlight the need for continued research and evaluation of malaria control interventions to assess their effectiveness and impact. Strengthening health systems, building partnerships, utilizing digital health technologies, and conducting context-specific research are recommended to improve healthcare access and reduce the burden of malaria in FCAS.

**Supplementary Information:**

The online version contains supplementary material available at 10.1007/s44197-023-00180-7.

## Introduction

Malaria continues to be a major global public health challenge, with estimated 247 million cases and 619,000 deaths reported in 2021 [1]. Despite significant progress in reducing malaria morbidity and mortality, the disease remains a major challenge in low- and middle-income countries (LMICs). Moreover, populations living in fragile and conflict-affected states (FCAS) are often the most vulnerable and at high risk of malaria due to factors, such as deteriorating healthcare systems, mass relocations, inadequate access to health care, limited infrastructure, and reduced resilience to shocks [2–4]. It should be noted that the goal of the Global Technical Strategy (GTS) for Malaria Elimination is a 90% reduction in malaria mortality and morbidity by 2030, with several countries (such as the Asia Pacific region), which have committed to eliminating malaria by 2030 [5]. However, the unique challenges posed by FCAS necessitate a more nuanced approach.

The objectives of reducing the disease burden and eliminating malaria are closely linked to the 3.3 Sustainable Development Goals (SDG). Although the presence of conflict and instability in FCAS complicates malaria control efforts by disrupting healthcare delivery, disrupting malaria control activities, and increasing the risk of transmission. The burden of malaria in FCAS is further exacerbated by other factors, such as nutrition status and poor living conditions. Additionally, the geopolitical volatility, uncertainty, and complexity of FCAS have instilled hesitancy for international cooperation. While there have been efforts made by individual countries to help these areas, health has been predominantly seen as a diplomatic tool rather than merely a humanitarian endeavour [6]. As a result, traditional malaria control interventions, such as bed nets, indoor residual spraying, and rapid diagnostic tests, may be less effective in these contexts [7].

**Fig. 1:**
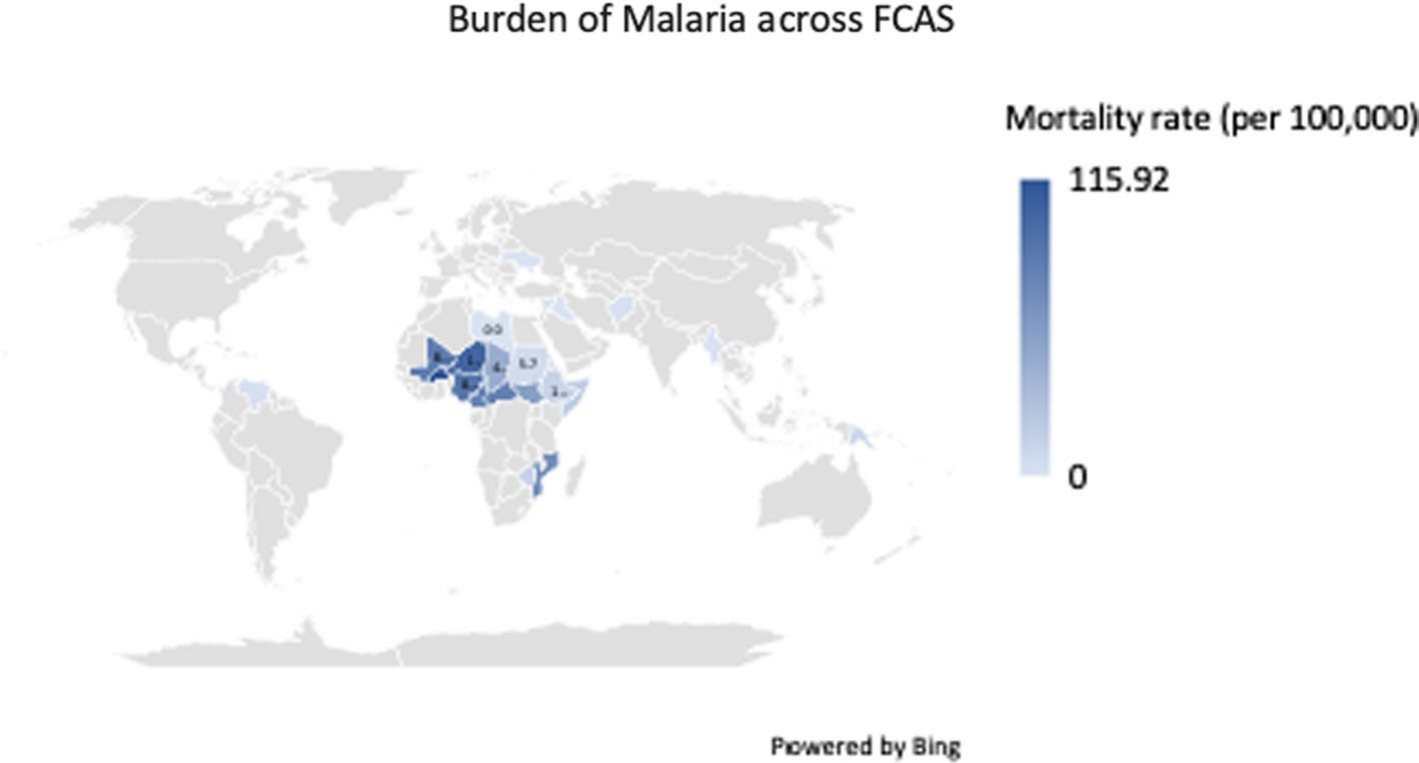
Burden of malaria across FCAS

Figure 1 illustrates the burden of malaria in FCAS in the year 2019, underscoring the heightened burden of the disease. Notable instances of escalated malaria prevalence in FCAS are observed in the Democratic Republic of the Congo, where conflict-stricken regions recorded a staggering 59% malaria prevalence in 2020 [1]. Similarly, in South Sudan, the rise in malaria cases can be attributed to conflict-induced environmental factors and shifts in climate, population displacement, worsening socioeconomic circumstances, limited availability of effective anti-malaria treatment, and the utilization of counterfeit anti-malarial drugs [8].

The rationale for this scoping review is grounded in the urgent need to address the complex and amplified malaria burden in Fragile and Conflict-Affected States (FCAS). In these settings, traditional malaria prevention and control measures often fall short due to the disrupted health systems and the unique challenges that arise from instability and conflict [7]. This study aims to bridge the gap in knowledge and intervention effectiveness by systematically identifying, organizing, and analyzing the emerging evidence tailored to the FCAS context [9]. The study aims to inform policy-makers and international health bodies, providing a comprehensive overview that can lead to more targeted, effective, and context-specific interventions. By doing so, it aligns with the broader global health objectives, such as the Sustainable Development Goals, and contributes to the strategic goal of a significant reduction in malaria morbidity and mortality, particularly in the most vulnerable populations. The findings of this scoping review could catalyse international cooperation and foster the development of innovative, evidence-based strategies that are essential for combating malaria in the most challenging environments.

## Methods

A scoping review was chosen to achieve the aim of this study as the primary purpose of scoping reviews is to identify and synthesise an existing or emerging body of literature on a given topic [10]. In this study, the authors followed the recommendations of the PRISMA extension for scoping reviews [11].

### Eligibility Criteria

#### Type of Studies

The articles for this review included randomized and non-randomized controlled studies, which are considered primary literature. Systematic reviews, editorials, views, and perspectives that did not include original research were excluded.

#### Type of Participants

Studies were included with participants of any age and gender.

#### Types of Interventions

We included trials that focussed on the malaria control interventions as suggested by WHO such as the Long Lasting Insecticidal Nets (LLIN), Indoor Residual Spraying (IRS), Larval Source Management (LSM)**,** Intermittent Preventive Treatment of pregnant women (IPTp), Intermittent Preventive Treatment in school-aged children (IPTsc), Perennial Malaria Chemoprevention (PMC), Post-Discharge Malaria Chemoprevention (PDMC), Mass Drug Administration (MDA), Targeted Drug Administration (TDA), Seasonal Malaria Chemoprevention (SMC), Intermittent Preventive Treatment for infants (IPTi), Rapid Diagnostic Tests (RDT), Artemisinin-based Combination Therapy (ACT), and Information, Education and Communication (IEC) campaigns [12].

#### Context

We included the studies that focussed on malaria elimination in FCAS. The FCAS, or the name of countries from the World Bank’s harmonized lists for 2018 and 2019, were considered [3]. The study included studies published from the launch of the World Health Organization's Roll Back Malaria (RBM) Partnership to End Malaria in 1998 through January 2023. Only studies published in English were included. The studies that are not in the context of FCASs listed by the World Bank and do not focus on malaria intervention were excluded.

#### Search Methods

A comprehensive search strategy was prepared using keywords, Boolean operators and with the use truncations. MEDLINE, EBSCO-CINAHL, Web of Science, ProQuest, and Cochrane Central Register of Controlled Trials were searched using specified search terms (Appendix 1). Reference lists of identified studies were also searched.

#### Selection of Studies

**Fig. 2:**
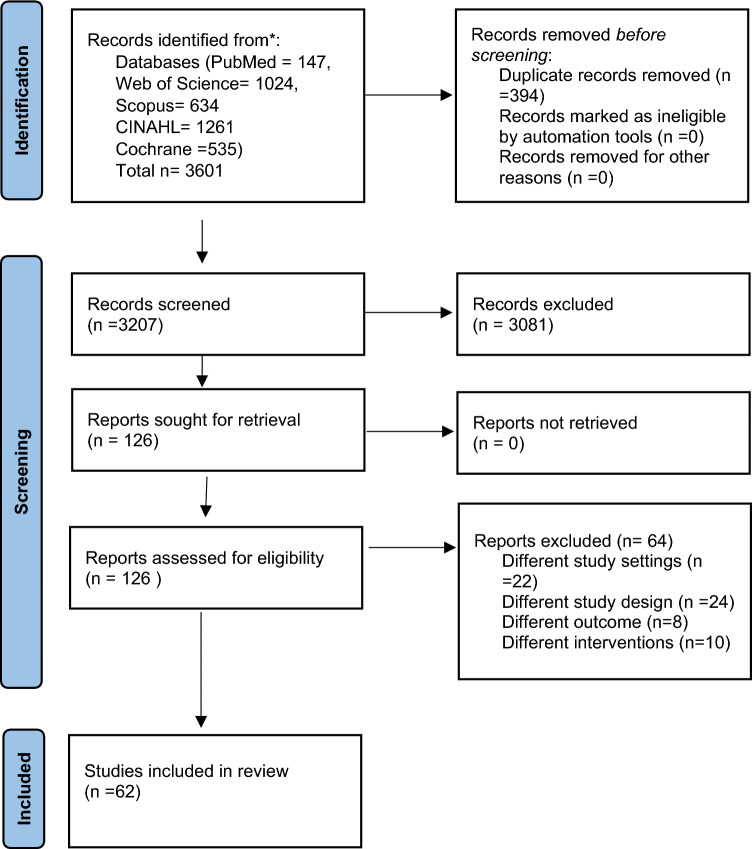
PRISMA flow diagram representing the study selection process [13]

Screening of the studies was carried out in two steps. First, two review authors independently assessed the titles and abstracts of trials identified by the searches to identify if the studies have met the inclusion criteria. During this stage, some of the trials were excluded as they did not meet the inclusion criteria. Second, the same review authors assessed the full texts of potentially relevant trials for inclusion using an eligibility form based on the inclusion criteria. More studies were excluded and the studies which met the inclusion criteria were included. Disagreements were resolved by discussion and consensus, with arbitration by the third review author if necessary. Finally, 62 articles were included in the review (Fig. 2).

#### Data Extraction and Management

A final set of records were imported into Rayyan, a web-based software program for screening, study selection, and data extraction. Two review authors independently extracted the information from the trials using pre-piloted, electronic data extraction forms. In case of differences in the extracted data, the two review authors discussed the differences to reach a consensus. If the issue remained unresolved, the third author engaged in further discussion.

#### Data Synthesis

The extracted data were summarized using narrative analysis. Studies were organized and described by the setting, population, sample, and the type of interventions adopted.

## Results

A total of 3601 studies were retrieved from a database search (PubMed-147, Web of science-1024, Scopus-634, CINAHL-1261, and Cochrane-535). After the screening, 62 studies that met the eligibility criteria were included in the synthesis. The detailed study selection is depicted in the PRISMA flowchart (Fig. 2). Characteristics of the included studies are presented in online supplementary document.

A total of 62 studies were included in this study, with six studies from fragile countries and 57 studies from conflict-affected countries. The fragile countries included Solomon Islands (*n* = 2), Papua New Guinea (*n* = 2), Guinea-Bissau (*n* = 1), and Comoros (*n* = 1). The conflict-affected countries included Afghanistan (*n* = 2), Burkina Faso (*n* = 18), Gabon (*n* = 6), Cameroon (*n* = 1, Democratic Republic of Congo (*n* = 1), Mali (*n* = 10), Mozambique, Myanmar (*n* = 4), and Nigeria (*n* = 8). The interventions reported in these countries targeted children below 5 years of age, those between 5 and 18 years of age, those between 18 and 60 years of age, pregnant women, and the general population.

**Fig. 3:**
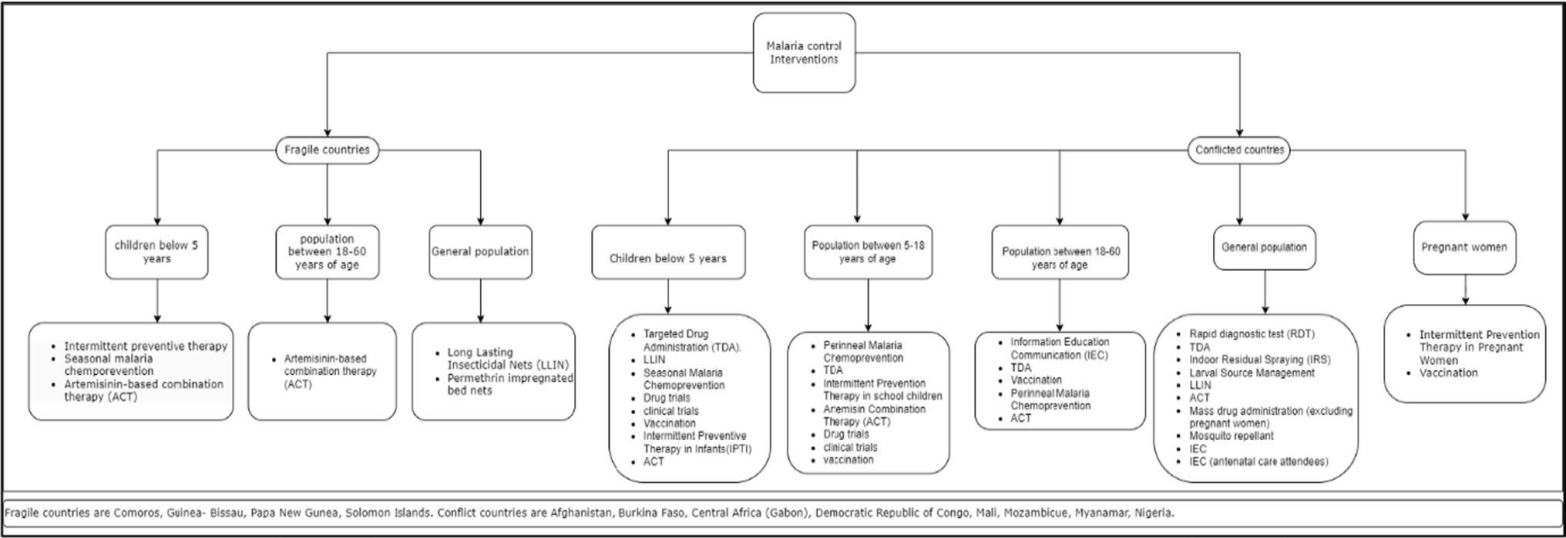
Malaria control interventions in FCAS countries

In fragile countries, the interventions reported for children below 5 years of age included IPTi (two studies in Papua New Guinea), TDA (one study from Guinea-Bissau), and ACT (one study from Comoros). For the population between 5 and 18 years of age and 18 and 60 years of age, ACT (one study from Comoros) was reported as an intervention. In the general population, LLINs and Permethrin-impregnated bed nets were reported as interventions, with both studies conducted in the Solomon Islands (Fig. 3).

Similarly, in conflict-affected areas, Interventions reported for children below 5 years of age included TDA (Four in Burkina Faso, one in Gabon and one in Mozambique), LLINs (three in Burkina Faso, one in the Democratic Republic of Congo), SMC (two in Burkina Faso, one in Mali), Clinical trials (two in Gabon and one in Nigeria), Malaria Vaccination (two in Burkina Faso, two in Mali, one in Mozambique, and one in Gabon), ACT (one in Nigeria), and IPTi (one in Gabon). For the population between 5 and 18 years of age, interventions reported were Perennial Malaria Chemoprevention (one in Myanmar), TDA (one in Burkina Faso, one in Gabon and one in Mali), IPT in School Children with ACT (one in Mali), Clinical trials (one in Burkina Faso and two in Gabon), and vaccination (one in Gabon). In the population between 18 and 60 years of age, interventions reported were IEC (one in Burkina Faso and one in Myanmar), TDA (one in Mali), Perennial Malaria Chemoprevention (one in Myanmar), ACT (one in Myanmar), and vaccination (two in Mali Interventions reported in pregnant women were IPT in pregnant women (one in Burkina Faso, one in Mozambique, and one in Mali), and vaccination (one in Burkina Faso). In the general population, interventions reported were RDTs (one in Afghanistan, one in Myanmar and three in Nigeria), TDA (one in Afghanistan and one in Cameroon), IRS one in Burkina Faso and one in Mozambique), Larval Source Management (two in Burkina Faso), LLINs (one in Mali, and one in Mozambique), ACT (one in Myanmar and one in Nigeria), Mosquito Repellent (one in Myanmar), and MDA (one in Myanmar). Antenatal Care Attendees received IEC as an intervention in Nigeria (Fig. 3).

## Discussion

Malaria presents a significant obstacle to public health in FCAS countries, highlighting the necessity for robust control measures to alleviate the impact of malaria outbreaks and support the execution of health programs and policies. Challenges are magnified in conflict zones, such as Sudan, where ongoing warfare has shattered the healthcare infrastructure since mid-April. The country has seen over 70% of hospitals compelled to suspend services, numerous facilities being bombed, and forced evacuations becoming commonplace, plunging the healthcare system into chaos, and leaving the populace extremely vulnerable. In resource-limited settings like these, adopting a comprehensive and collaborative approach to comprehend the contextual differences in implementing interventions in FCAS becomes particularly relevant [21].

On the global stage, despite various conflicts, there is a history of international cooperation towards shared goals, notably seen in the pursuit of the SDG 3—Good Health and Well-Being gaining prominence during the COVID-19 pandemic. While health issues have been prioritized in global dialogues, efforts to eliminate malaria in FCAS have lagged. The presence of malaria can deter foreign investments and the establishment of embassies, which are vital for diplomatic activities and disease monitoring—critical roles in FCAS, where the majority of infectious disease outbreaks occur. Lack of diplomatic presence exacerbates conflict risks and disease spread, leading to economic and political instability. Study by Bagozzi et al. indicates that malaria is a significant determinant of diplomatic relations between two countries, as higher malaria rates increase the hesitancy of countries to interact with the affected region. For example, malaria was also one of the major concerns for late Ottoman state and society, and malaria regulations at times encouraged the establishment of enormous estates in the countryside of the Mediterranean littoral [22]. Malaria burden was increased in Korea during the Japanese colonial rule. Japanese anti-malarial efforts focussed on military garrisons in rural and urban regions, at the expense of both civilian settlers and Koreans. However, Koreans faced the brunt of the malaria epidemic, which was worsened in many regions by agricultural and industrial expansion, and, eventually, by the military government established in 1938 [23]. This highlights the need for a holistic approach to the SDGs, recognising that addressing health challenges like malaria requires the involvement of multiple sectors beyond healthcare [24, 25].

The current review shows 62 malaria control intervention trials across the FCAS countries. Some studies suggest that malaria interventions have been maintained in FCAS despite ongoing conflicts [26–28]. This finding is congruent with our study. However, a persistent high burden of malaria has been reported following the scale-up of malaria control interventions. Therefore, it is necessary to continue monitoring and evaluating malaria control programs to ensure sustained effectiveness and identify improvement areas. The GTS 2016–2030 advocates for two sets of interventions: vector control-based prevention, diagnosis, and prompt effective treatment of malaria cases. The GTS has the ambitious goal of malaria elimination from at least 35 countries by 2030 [29].

In this paper, the existing identified interventions in FCAS have been categorised based on age. Several studies have evaluated the impact of these interventions on malaria morbidity and mortality. Several studies have evaluated the impact of these interventions on malaria morbidity and mortality. Bhattarai et al*.* report that high coverage of combined malaria control interventions can reduce the malaria burden in tropical Africa and achieve the SDGs of reducing mortality in children under five and alleviating the burden of malaria [14]. The findings of the study by Kayentao complements our study findings as the observed decrease in all-cause child mortality and morbidity aligned with the timing of the expansion of malaria control interventions in Mali. This entailed the nationwide distribution of ITNs and the adoption of ACTs as the primary treatment for malaria [15]. Additionally, using LLINs effectively reduces malaria parasitaemia in children under 5 years of age [30]. However, there is still a wide variation in care-seeking practices for children under five with fever across countries. This indicates the need for continued efforts to improve access to and utilization of malaria control interventions [31].

Measures for 5–18 year age group identified in the study have been proven successful [32–34]. Furthermore, Tiono et al*.* compared active and passive case detection methods to determine malaria incidence among children in Burkina Faso. The study emphasizes the continuing need for enhanced efforts in malaria surveillance and diagnosis [16]. Moreover, there is still a wide variation in care-seeking practices for children under five with fever across countries. This indicates the need for continued efforts not only to improve access to and utilization of malaria control interventions but also to address help seeking behaviours risk awareness and risk mitigation practices. These studies have also found that the campaign significantly increased bed net ownership and use, reducing malaria morbidity and mortality in the general population and in pregnant women [17–20].

However, approaches to expand malaria control interventions in areas of active conflict are urgently needed [35]. To achieve the targets of GTS, further steps need to be taken in accordance with the strategy to prevent malaria transmission [36]. The RBM Partnership in the Democratic Republic of Congo (DRC) and the Malaria Elimination Task Force (METF) in Myanmar are examples of a comprehensive approach to malaria elimination in an FCAS. These partnerships involve collaboration between the government, NGOs, UN agencies (WHO, UNDP, UNICEF, and the World Bank), and the private sector to reduce the burden of malaria. These partnerships focus on strengthening health systems, increasing access to malaria prevention and treatment services, and engaging communities in malaria control efforts [37, 38].

Additionally, the inherent fragility of the health system due to unstable and uncertain circumstances can hinder people living in FCAS from gaining access to healthcare such as malaria interventions. Therefore, we need suitable preventive measures and region-specific health interventions to understand better the unique dynamics of the disease process within FCAS. Several strategies can be implemented to enhance healthcare access in FCAS, specifically for malaria prevention. One effective approach is to build local partnerships and capacities, utilizing local resources to provide healthcare services to vulnerable populations. An example is the MENTOR initiative, established in 2002 by Richard Allan. By training and supporting Community Health Workers (CHWs), MENTOR delivers primary healthcare to displaced, vulnerable, and inaccessible communities. These CHWs are mobile and can offer continuous medical care, particularly for malaria, diarrhoea, and respiratory diseases. This initiative is currently active in Mozambique, Angola, Syria, and Venezuela [39].

Digital health technologies also play a crucial role in improving healthcare access in FCAS. By utilizing appropriate and tailored digital tools, the quality, accessibility, and availability of healthcare services can be enhanced [40]. This can include remote diagnosis and treatment, facilitating access to malaria prevention interventions. The role of such technologies has been tested and proven in the form of digital surveillance and case management systems [41], spatial mapping [42, 43], and standardised EMS systems in FCAS LMIC settings [44].

**Fig. 4:**
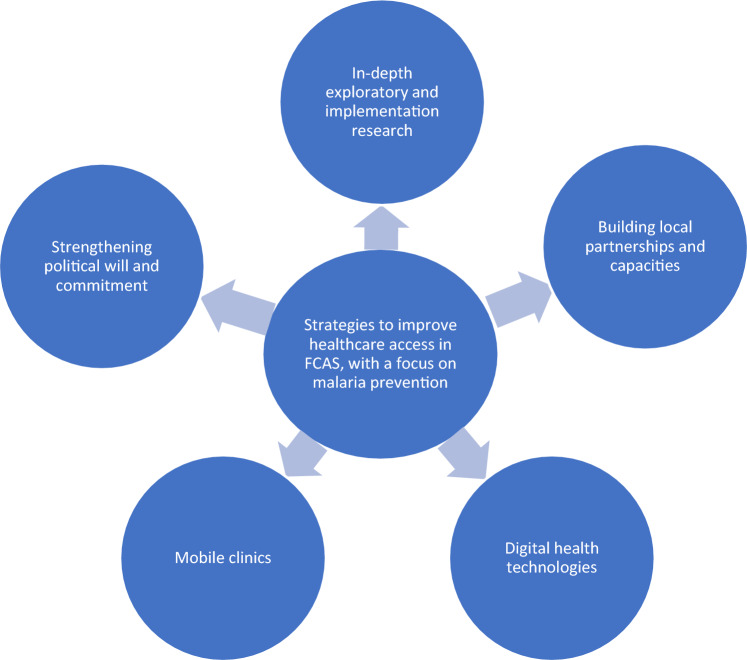
Strategies to improve healthcare access in FCAS, with a focus on malaria prevention

Mobile clinics present another viable model of care for malaria prevention interventions in FCAS. These clinics can be deployed to ensure the continuum of malaria care and treatment, reaching communities that may otherwise have limited access to healthcare services [45]. A critical aspect of improving healthcare access in FCAS is strengthening political will and commitment. By securing and reinforcing political support, prioritization of malaria prevention interventions can be achieved, leading to increased accessibility of healthcare services. Furthermore, in-depth exploratory and implementation research is necessary to shape and test malaria prevention interventions in FCAS. Conducting context-specific studies allows the development of effective and tailored malaria prevention strategies that address the unique challenges faced in FCAS. This research can inform the implementation of targeted interventions to improve healthcare access and reduce the burden of malaria in these settings [46] (Fig. 4).

## Conclusion

Understanding the interventions in FCAS and developing a context-specific intervention to prevent malaria is vital. The study provides valuable insights into the interventions used to control malaria in fragile and conflicted countries. The interventions reported in the review are diverse and cover a wide range of population groups. The review also highlights the need for continued monitoring and evaluation of malaria control interventions to assess their effectiveness and impact. The studies reported in the review provide evidence for the effectiveness of various interventions in reducing the burden of malaria in different population groups. The review's findings can be used to inform policy-makers and the international health bodies on the development of targeted interventions to reduce the burden of malaria in these settings. The review also highlights the need for continued research and evaluation of malaria control interventions to assess their effectiveness and impact.

Despite the efforts by the international community to fight malaria, it continues to be endemic while imposing a greater threat to FCAS. The global players need to ensure that malaria is not a diplomatic tool that can be used to leverage its geopolitical power. It is crucial for countries that operate in silos and competitively without cooperation to recognise that it would be difficult for the world to meet the SDG 3 goals. Thus, we must address global health challenges such as malaria through partnerships (SDG 17) to ensure a world with peace, justice, and resilient institutions (SDG 16).

### Supplementary Information

Below is the link to the electronic supplementary material.

Supplementary file1 (DOCX 426 KB)

## Data Availability

All data underlying the results are available as part of the article and no additional source data are required.

## Abbreviations

ACT: Artemisinin-based Combination Therapy
CHW: Community Health Workers
DRC: Democratic Republic of Congo
FCAS: Fragile and Conflict-Affected States
GTS: Global Technical Strategy
IEC: Information, Education and Communication
IPTi: Intermittent Preventive Treatment for infants
IPTp: Intermittent Preventive Treatment of pregnant women
IPTsc: Intermittent Preventive Treatment in school-aged children
IRS: Indoor Residual Spraying
LLIN: Long Lasting Insecticidal Nets
LMIC: Low- and Middle-Income Countries
LSM: Larval Source Management
MDA: Mass Drug Administration
METF: Malaria Elimination Task Force
PDMC: Post-Discharge Malaria Chemoprevention
PMC: Perennial Malaria Chemoprevention
RBM: Roll Back Malaria
RDT: Rapid Diagnostic Tests
SDG: Sustainable Development Goal
SMC: Seasonal Malaria Chemoprevention
TDA: Targeted Drug Administration
UNDP: United Nations Development Programme
UNICEF: United Nations International Children's Emergency Fund
WHO: World Health Organization
