# Supplementary material for: A Scoping Review on Malaria Prevention and Control Intervention in Fragile and Conflict-Affected States (FCAS): A Need for Renewed Focus to Enhance International Cooperation
Source: J Epidemiol Glob Health. 2024 Jan 15;14(1):4–12. doi: 10.1007/s44197-023-00180-7 (PMC11043240; doi:10.1007/s44197-023-00180-7)
Supplement: Supplementary file 1 — Supplementary file1 (DOCX 426 KB) [file 44197_2023_180_MOESM1_ESM.docx]

## **Appendix 1: Supplementary materials**

Table of Contents

[Appendix 1: Supplementary materials 1](#_Toc138403740)

[Table 1: Search strategy 2](#_Toc138403741)

[Table 2: Characteristics of the included studies 3](#_Toc138403742)

## **Table 1: Search strategy**

| MEDLINE | "Malaria"[MeSH Major Topic] OR "Malaria"[Title/Abstract] OR "infections plasmodium"[Text Word] OR "Plasmodium Infection"[Text Word] OR "Remittent Fever"[Text Word] OR "Plasmodium Infections"[Text Word] AND "malaria/prevention and control"[MeSH Terms] OR mosquito control [Mesh] OR malaria control [Text Word] OR "Long lasting insecticidal nets"[Text Word] OR "LLIN"[Text Word] OR "indoor residual spraying"[Text Word] OR "IRS"[Text Word] OR "Larval source management"[Text Word] OR "LSM"[Text Word] OR "IPTp"[Text Word] OR "IPTsc"[Text Word] OR "PMC"[Text Word] OR "Post-discharge malaria chemoprevention"[Text Word] OR "PDMC"[Text Word] OR "Mass drug administration"[Text Word] OR "MDA"[Text Word] OR "Targeted drug administration"[Text Word] OR "TDA"[Text Word] OR "seasonal malaria chemoprevention"[Text Word] OR "SMC"[Text Word] OR "IPTi"[Text Word] OR "RDT"[Text Word] OR "artemisinin-based combination therapy"[Text Word] OR "ACT"[Text Word] OR "Information Education and Communication"[Text Word] OR "IEC"[Text Word] AND "Fragile and conflict affected settings"[Title/Abstract] OR "Fragile and conflict affected settings"[Text Word] OR "FCAS"[Text Word] OR ((("Afghanistan"[Text Word] OR "Armenia"[Text Word] OR "Azerbaijan"[Text Word] OR "Burkina"[Text Word] OR "Faso"[Text Word] OR "Cameroon"[Text Word] OR "Central"[Text Word] OR "African"[Text Word] OR "Republic"[Text Word] OR "Congo"[Text Word] OR "Democratic Republic of Ethiopia"[All Fields] OR "Iraq"[Text Word] OR "Mali"[Text Word] OR "Mozambique"[Text Word] OR "Myanmar"[Text Word] OR "Niger"[Text Word] OR "Nigeria"[Text Word] OR "Somalia"[Text Word] OR "South Sudan"[Text Word] OR "Syrian Arab Republic"[Text Word] OR "Ukraine"[Text Word] OR "Burundi"[Text Word] OR "Chad"[Text Word] OR "Comoros"[Text Word] OR "congo republic"[Text Word] OR "Eritrea"[Text Word] OR "Guinea-Bissau"[Text Word] OR "Haiti"[Text Word] OR "Kosovo"[Text Word] OR "Lebanon"[Text Word] OR "Libya"[Text Word] OR "Marshall Islands"[Text Word] OR "micronesia federated states of"[Text Word] OR "Papua New Guinea"[Text Word] OR "Solomon Islands"[Text Word] OR "Sudan"[Text Word] OR "Timor-Leste"[Text Word] OR "Tuvalu"[Text Word] OR "West Bank and Gaza"[Text Word]) AND "West Bank and Gaza"[All Fields]) OR ("zimbabwe"[MeSH Terms] OR "zimbabwe"[All Fields] OR "Zimbabwe*"[All Fields])) |
| --- | --- |
| Web of Science | TI=("malaria/prevention and control" OR mosquito control OR malaria control OR "Long lasting insecticidal nets" OR "LLIN" OR "indoor residual spraying" OR "IRS" OR "Larval source management" OR "LSM" OR "IPTp" OR "IPTsc" OR "PMC" OR "Post-discharge malaria chemoprevention” OR "PDMC" OR "Mass drug administration" OR "MDA" OR "Targeted drug administration" OR "TDA" OR "seasonal malaria chemoprevention" OR "SMC" OR "IPTi" OR "RDT" OR "artemisinin-based combination therapy" OR "ACT" OR "Information Education and Communication" OR "IEC" ) AND ALL=(“Fragile and conflict affected settings“ OR "FCAS" OR “Afghanistan“ OR “Armenia“ OR “Azerbaijan“ OR “Burkina“ OR “Faso“ OR “Cameroon“ OR “Central“ OR “African“ OR “Republic Congo“ OR “Democratic Republic of Ethiopia“ OR “Iraq“ OR “Mali“ OR Mozambique OR Myanmar OR Niger OR Nigeria OR Somalia OR South Sudan OR Syrian Arab Republic OR Ukraine OR Burundi OR Chad OR Comoros OR congo republic OR Eritrea OR Guinea-Bissau OR Haiti OR Kosovo OR Lebanon OR Libya OR Marshall Islands OR “micronesia federated states of” OR Papua New Guinea OR Tuvalu OR Solomon Islands OR Sudan OR Timor-Leste OR “Zimbabwe“ OR "West Bank and Gaza") NOT ALL=(incidence OR prevalence) AND TI=("Malaria” OR "Malaria” OR "infections plasmodium" OR "Plasmodium Infection” OR "Remittent Fever" OR "Plasmodium Infections") |
| Cochrane Central Register of Controlled Trials | ("Malaria" OR "infections plasmodium" OR "Plasmodium Infection" OR "Remittent Fever" OR "Plasmodium Infections") AND ("malaria prevention" OR "mosquito control" OR "malaria control" OR "Long lasting insecticidal nets" OR "LLIN" OR "indoor residual spraying" OR "IRS" OR "Larval source management" OR "LSM" OR "IPTp" OR "IPTsc" OR "PMC" OR "Post-discharge malaria chemoprevention" OR "PDMC" OR "Mass drug administration" OR "MDA" OR "Targeted drug administration" OR "TDA" OR "seasonal malaria chemoprevention" OR "SMC" OR "IPTi" OR "RDT" OR "artemisinin-based combination therapy" OR "ACT" OR "Information Education and Communication" OR "IEC") AND ("Fragile and conflict-affected settings" OR "Fragile and conflict affected settings" OR "FCAS" OR "Afghanistan" OR "Armenia" OR "Azerbaijan" OR "Burkina" OR "Faso" OR "Cameroon" OR "Central African Republic" OR "Congo" OR "Democratic Republic of Ethiopia" OR "Iraq" OR "Mali" OR "Mozambique" OR "Myanmar" OR "Niger" OR "Nigeria" OR "Somalia" OR "South Sudan" OR "Syrian Arab Republic" OR "Syria" OR "Ukraine" OR "Burundi" OR "Chad" OR "Comoros" OR "congo republic" OR "Eritrea" OR "Guinea Bissau" OR "Haiti" OR "Kosovo" OR "Lebanon" OR "Libya" OR "Marshall Islands" OR "micronesia federated states of" OR "Papua New Guinea" OR "Solomon Islands" OR "Sudan" OR "Timor-Leste" OR "Tuvalu" OR "West Bank and Gaza" OR "Zimbabwe") |
| EBSCO-CINAHL | "TI ( "Malaria" OR "infections plasmodium" OR "Plasmodium Infection"OR "Remittent Fever" OR "Plasmodium Infections" ) OR AB ( "Malaria" OR "infections plasmodium" OR "Plasmodium Infection"OR "Remittent Fever" OR "Plasmodium Infections" ) AND "TI ( "malaria/prevention and control" OR mosquito control OR malaria control OR "Long lasting insecticidal nets" OR "LLIN" OR "indoor residual spraying" OR "IRS" OR "Larval source management"OR "LSM"OR "IPTp"OR "IPTsc"OR "PMC" OR "Post-discharge malaria chemoprevention"OR "PDMC" OR "Mass drug administration"OR "MDA" OR "Targeted drug administration" OR "TDA" OR "seasonal malaria chemoprevention" OR "SMC"OR "IPTi" OR "RDT" OR "artemisinin-based combination therapy" OR "ACT" OR "Information Education and Communication” OR "IEC" ) OR AB ( "malaria/prevention and control" OR mosquito control OR malaria control OR "Long lasting insecticidal nets" OR "LLIN" OR "indoor residual spraying" OR "IRS" OR "Larval source management"OR "LSM"OR "IPTp"OR "IPTsc"OR "PMC" OR "Post-discharge malaria chemoprevention"OR "PDMC" OR "Mass drug administration"OR "MDA" OR "Targeted drug administration" OR "TDA" OR "seasonal malaria chemoprevention" OR "SMC"OR "IPTi" OR "RDT" OR "artemisinin-based combination therapy" OR "ACT" OR "Information Education and Communication” OR "IEC" ) AND "TI ( "malaria/prevention and control" OR mosquito control OR malaria control OR "Long lasting insecticidal nets" OR "LLIN" OR "indoor residual spraying" OR "IRS" OR "Larval source management"OR "LSM"OR "IPTp"OR "IPTsc"OR "PMC" OR "Post-discharge malaria chemoprevention"OR "PDMC" OR "Mass drug administration"OR "MDA" OR "Targeted drug administration" OR "TDA" OR "seasonal malaria chemoprevention" OR "SMC"OR "IPTi" OR "RDT" OR "artemisinin-based combination therapy" OR "ACT" OR "Information Education and Communication” OR "IEC" ) OR AB ( "malaria/prevention and control" OR mosquito control OR malaria control OR "Long lasting insecticidal nets" OR "LLIN" OR "indoor residual spraying" OR "IRS" OR "Larval source management"OR "LSM"OR "IPTp"OR "IPTsc"OR "PMC" OR "Post-discharge malaria chemoprevention"OR "PDMC" OR "Mass drug administration"OR "MDA" OR "Targeted drug administration" OR "TDA" OR "seasonal malaria chemoprevention" OR "SMC"OR "IPTi" OR "RDT" OR "artemisinin-based combination therapy" OR "ACT" OR "Information Education and Communication” OR "IEC" ) |
| Proquest | title("Malaria" OR "Malaria" OR "infections plasmodium" OR "Plasmodium Infection" OR "Remittent Fever" OR "Plasmodium Infections") AND title("malaria/prevention and control" OR mosquito control OR malaria control OR "Long lasting insecticidal nets" OR "LLIN" OR "indoor residual spraying" OR "IRS" OR "Larval source management"OR "LSM"OR "IPTp"OR "IPTsc"OR "PMC" OR "Post-discharge malaria chemoprevention"OR "PDMC" OR "Mass drug administration"OR "MDA" OR "Targeted drug administration" OR "TDA" OR "seasonal malaria chemoprevention" OR "SMC"OR "IPTi" OR "RDT" OR "artemisinin-based combination therapy" OR "ACT" OR "Information Education and Communication” OR "IEC" ) AND title("Fragile and conflict affected settings" OR "Fragile and conflict affected settings" OR "FCAS" ) OR abstract(Afghanistan OR Armenia OR Azerbaijan OR Burkina OR Faso OR Cameroon OR Central OR "African "Republic” OR "Congo" OR "Democratic Republic of Ethiopia" OR Iraq OR Mali OR "Mozambique" OR "Myanmar" OR "Niger" OR Nigeria OR "Somalia OR "South Sudan" OR "Syrian Arab Republic" OR Ukraine OR Burundi OR Chad OR Comoros OR congo republic OR Eritrea OR Guinea-Bissau OR Haiti OR Kosovo OR Lebanon OR Libya OR "Marshall Islands"OR "micronesia federated states of" OR "Papua New Guinea" OR "Solomon Islands" OR Sudan OR "Timor-Leste" OR Tuvalu OR "West Bank and Gaza" OR "West Bank and Gaza" OR zimbabwe) |

## **Table 2: Characteristics of the included studies**

| **Sl. No.** | **Author, Year** | **Country** | **Title of the study** | **Population** | **Interventions** |
| --- | --- | --- | --- | --- | --- |
| 1 | Guoming li, 2022 ​[1] | Comoros | Artemisinin-piperaquine versus artemether-lumefantrine for treatment of uncomplicated *Plasmodium falciparum* malaria in Grande Comore Island: an open-label, non-randomised controlled trial | Age 2-60 years | AP vs AL treatment (ACT) |
| 2 | P E Kofoed, 2011 ​​[2] | Guinea- Bissau | Paracetamol versus placebo in treatment of non-severe malaria in children in Guinea-Bissau: a randomized controlled trial | children | Targeted Drug Administration (TDA) |
| 3 | C Pell, 2010 ​[3] | Papua New Guinea | Community response to intermittent preventive treatment of malaria in infants (IPTi) in Papua New Guinea | Infants | Intermittent Preventive Treatment |
| 4 | O Sottas, 2019[4] ​​ | Papua New Guinea | Adherence to intermittent preventive treatment for malaria in Papua New Guinean infants: A pharmacological study along side the randomized controlled trial | Children | Intermittent Preventive Treatment |
| 5 | P M Mankadi, 2021[5]​ | Democratic Republic of Congo | Effects of Door-to-Door Hang-Up Visits on the Use of Long-Lasting Insecticide-Treated Mosquito Nets in the Democratic Republic of the Congo: A Cluster Randomized Controlled Trial | Children under 5 years of age | Long Lasting Insecticidal Nets (LLIN) |
| 6 | Atkinson, 2009[6] ​​ | Solomon Islands | A cluster randomized controlled cross-over bed net acceptability and preference trial in Solomon Islands: community participation in shaping policy for malaria elimination. | General Population | Long Lasting Insecticidal Nets (LLIN) |
| 7 | Kere, 1996[7] | Solomon Islands | Permethrin-impregnated bed nets are more effective than DDT house-spraying to control malaria in Solomon Islands | General Population | Permethrin Impregnated bed nets |
| 8 | Leslie, 2017 ​[8] | Afghanistan | Use of malaria rapid diagnostic tests by community health workers in Afghanistan: cluster randomised trial. | General Population | Rapid Diagnostic Tests (RDT) |
| 9 | Leslie, 2007 ​[9] | Afghanistan | Sulfadoxine-Pyrimethamine, Chlorproguanil-Dapsone, or Chloroquine for the Treatment of Plasmodium vivax Malaria in Afghanistan and Pakistan | General Population with confirmed P Vivax malaria | Targeted Drug Administration (TDA) |
| 10 | DD Soma, 2021 ​​[10] | Burkina Faso | Insecticide resistance status of malaria vectors *Anopheles gambiae* (*s.l.*) of southwest Burkina Faso and residual efficacy of indoor residual spraying with microencapsulated pirimiphos-methyl insecticide | General Population | Indoor Residual Spraying (IRS) |
| 11 | Kouyaté B, 2008 [11]​​ | Burkina Faso | Process and effects of a community intervention on malaria in rural Burkina Faso: randomized controlled trial | Age 18-60 years | Information Education Communication (IEC) |
| 12 | Tagbor, 2015 [12]​​ | Burkina Faso | A Non-Inferiority, Individually Randomized Trial of Intermittent Screening and Treatment versus Intermittent Preventive Treatment in the Control of Malaria in Pregnancy | Pregnant Women | Intermittent Preventive Treatment |
| 13 | Bassat Q, 2009 ​​[13] | Burkina Faso | Dihydroartemisinin-Piperaquine and Artemether-Lumefantrine for Treating Uncomplicated Malaria in African Children: A Randomised, Non-Inferiority Trial | Children under 5 years of age | Targeted Drug Administration (TDA) |
| 14 | J Brogdon, 2022 ​​[14] | Burkina Faso | Malaria positivity following a single oral dose of azithromycin among children in Burkina Faso: a randomized controlled trial | Children under 5 years of age | Targeted Drug Administration (TDA) |
| 15 | V R Louis, 2012 [15]​ | Burkina Faso | Long-term effects of malaria prevention with insecticide-treated mosquito nets on morbidity and mortality in African children: randomised controlled trial | under 5 years of age | Long Lasting Insecticidal Nets (LLIN) |
| 16 | Coulibaly, 2009[16] ​​ | Burkina Faso | Strong Gametocytocidal Effect of Methylene Blue-Based Combination Therapy against Falciparum Malaria: A Randomised Controlled Trial | under 5 years of age | Seasonal Malaria Chemoprevention |
| 17 | Muller O, 2006 [17]​ | Burkina Faso | Effects of insecticide-treated bed nets during early infancy in an African area of intense malaria transmission: a randomized controlled trial | under 5 years of age | Long Lasting Insecticidal Nets (LLIN) |
| 18 | Dambach P, 2020 [18]​ | Burkina Faso | Biological larviciding against malaria vector mosquitoes with Bacillus thuringiensis israelensis (Bti) – Long term observations and assessment of repeatability during an additional intervention year of a large-scale field trial in rural Burkina Faso | General Population | Larval Source management |
| 19 | Sirima S B, 2020 ​​[19] | Burkina Faso | PRIMVAC vaccine adjuvanted with Alhydrogel or GLA-SE to prevent placental malaria: a first-in-human, randomised, double-blind, placebo-controlled study | Pregnant Women | Vaccination |
| 20 | Tiono AB, 2018[20] ​​ | Burkina Faso | Efficacy of Olyset Duo, a bednet containing pyriproxyfen and permethrin, versus a permethrin-only net against clinical malaria in an area with highly pyrethroid-resistant vectors in rural Burkina Faso: a cluster-randomised controlled trial | under 5 years of age | Long Lasting Insecticidal Nets (LLIN) |
| 21 | Zeba AN, 2008 ​[21] | Burkina Faso | Major reduction of malaria morbidity with combined vitamin A and zinc supplementation in young children in Burkina Faso: a randomized double-blind trial | Age 6 months - 6 years | Targeted Drug Administration (TDA) |
| 22 | Dambach P, 2019 [22] | Burkina Faso | Reduction of malaria vector mosquitoes in a large-scale intervention trial in rural Burkina Faso using Bti based larval source management | General Population | Larval Source management |
| 23 | Zongo I, 2015[23] | Burkina Faso | Randomized Noninferiority Trial of Dihydroartemisinin-Piperaquine Compared with Sulfadoxine-Pyrimethamine plus Amodiaquine for Seasonal Malaria Chemoprevention in Burkina Faso | under 5 years of age | Seasonal Malaria Chemoprevention |
| 24 | Zoungrana A, 2008[24] ​ | Burkina Faso | Safety and Efficacy of Methylene Blue Combined with Artesunate or Amodiaquine for Uncomplicated Falciparum Malaria: A Randomized Controlled Trial from Burkina Faso | Age 5-18 years | Clinical trials |
| 25 | Coulibaly, 2021 ​[25] | Burkina Faso | Effect of a single dose of oral azithromycin on malaria parasitaemia in children: a randomized controlled trial | under 5 years of age | Targeted Drug Administration (TDA) |
| 26 | D Chandramohan, 2021 ​​[26] | Burkina Faso | Seasonal Malaria Vaccination with or without Seasonal Malaria Chemoprevention | Children under 5 years of age | Vaccination |
| 27 | Datoo Ms, 2021 ​​[27] | Burkina Faso | Efficacy of a low-dose candidate malaria vaccine, R21 in adjuvant Matrix-M, with seasonal administration to children in Burkina Faso: a randomised controlled trial | Age 0-5 years of age | Vaccination |
| 28 | Mbacham, 2014 [28]​​ | Cameroon | Basic or enhanced clinician training to improve adherence to malaria treatment guidelines: a cluster-randomised trial in two areas of Cameroon. | General Population | Targeted Drug Administration |
| 29 | Nsimba, 2008 [29] ​​ | Gabon | Sulphadoxine/pyrimethamine versus amodiaquine for treating uncomplicated childhood malaria in Gabon: A randomized trial to guide national policy | under 5 years of age | Targeted Drug Administration |
| 30 | Grobusch, 2007 [30]​​ | Gabon | Intermittent Preventive Treatment against Malaria in Infants in Gabon—A Randomized, Double-Blind, Placebo-Controlled Trial | under 5 years of age | Intermittent Preventive Treatment |
| 31 | Oyakhirome S, 2007[31] ​ | Gabon | Randomized Controlled Trial of Fosmidomycin- Clindamycinversus Sulfadoxine-Pyrimethamine in the Treatment of Plasmodium falciparum Malaria | Age 5-18 years | Clinical trials |
| 32 | Kremsner P, 2016 [32]​ | Gabon | Intramuscular Artesunate for Severe Malaria in African Children: A Multicentre Randomized Controlled Trial | under 5 years of age/ Age 5-18 years | Clinical trials |
| 33 | A Alloueche, 2004 [33]​ | Gabon | Comparison of chlorproguanil-dapsone with sulfadoxine pyrimethamine for the treatment of uncomplicated falciparum malaria in young African children: double-blind randomised controlled trial | under 5 years of age | Clinical trials |
| 34 | S Belard, 2011 [34] ​​ | Gabon | A Randomized Controlled Phase Ib Trial of the Malaria Vaccine Candidate GMZ2 in African Children | Age 5-18 years | Vaccination |
| 35 | Dicko, 2011 [35]​​ | Mali | Increase in EPI vaccines coverage after implementation of intermittent preventive treatment of malaria in infant with Sulfadoxine-pyrimethamine in the district of Kolokani, Mali: Results from a cluster randomized control trial | under 5 years of age | Vaccination |
| 36 | Dicko, 2016 ​​[36] | Mali | Primaquine to reduce transmission of Plasmodium falciparum malaria in Mali: a single-blind, dose-ranging, adaptive randomised phase 2 trial | Age 5-18 years/ age 18-60 years | Targeted Drug Administration |
| 37 | Cairns ME, 2020 ​​[37] | Mali | Evaluation of seasonal malaria chemoprevention in two areas of intense seasonal malaria transmission: Secondary analysis of a household-randomised, placebo-controlled trial in Houndé District, Burkina Faso and Bougouni District, Mali | under 5 years of age | Seasonal Malaria Chemoprevention |
| 38 | D Chandramohan, 2021[38] ​​ | Mali | Seasonal Malaria Vaccination with or without Seasonal Malaria Chemoprevention | Children under 5 years of age | Vaccination |
| 39 | Maiga OM, 2011[39] ​ | Mali | Superiority of 3 Over 2 Doses of Intermittent Preventive Treatment With Sulfadoxine-Pyrimethamine for the Prevention of Malaria During Pregnancy in Mali: A Randomized Controlled Trial | Pregnant Women | Intermittent Preventive Treatment |
| 40 | Maiga H, 2022 ​[40] | Mali | Overall and Gender-Specific Effects of Intermittent Preventive Treatment of Malaria with Artemisinin-Based Combination Therapies among Schoolchildren in Mali: A Three-Group Open Label Randomized Controlled Trial | age 5-18 years | Intermittent Preventive Treatment in School Children with ACT |
| 41 | Rhee, 2005 ​​ [41] | Mali | Use of insecticide-treated nets (ITNs) following a malaria education intervention in Piron, Mali: a control trial with systematic allocation of households | General Population | Long Lasting Insecticidal Nets (LLIN) |
| 42 | Sissoko MS, 2022 ​[42] | Mali | Safety and efficacy of a three-dose regimen of Plasmodium falciparum sporozoite vaccine in adults during an intense malaria transmission season in Mali: a randomised, controlled phase 1 trial | Age 18-60 years | Vaccination |
| 43 | Sagara I, 2009 ​[43] | Mali | A randomized and controlled Phase 1 study of the safety and immunogenicity of the AMA1-C1/Alhydrogel® + CPG 7909 vaccine for Plasmodium falciparum malaria in semi-immune Malian adults | Age 18-60 years | Vaccination |
| 44 | Barry A, 2018 ​[44] | Mali | Optimal mode for delivery of seasonal malaria chemoprevention in Ouelessebougou, Mali: A cluster randomized trial | under 5 years of age | Seasonal Malaria Chemoprevention |
| 45 | Bassat Q, 2009 [45]​ | Mozambique | Dihydroartemisinin-Piperaquine and Artemether-Lumefantrine for Treating Uncomplicated Malaria in African Children: A Randomised, Non-Inferiority Trial | Children under 5 years of age | Targeted Drug Administration |
| 46 | Aponte JJ, 2007 ​[46] | Mozambique | Safety of the RTS,S/AS02D candidate malaria vaccine in infants living in a highly endemic area of Mozambique: a double blind randomised controlled phase I/IIb trial | under 5 years of age | Vaccination |
| 47 | Menendez, 2016 [47] ​ | Mozambique | Malaria Prevention with IPTp during Pregnancy Reduces Neonatal Mortality | Pregnant Women | Intermittent Preventive Treatment in pregnant women |
| 48 | Wagman JM, 2021 ​[48] | Mozambique | Reduced exposure to malaria vectors following indoor residual spraying of pirimiphos-methyl in a high-burden district of rural Mozambique with high ownership of long-lasting insecticidal nets: entomological surveillance results from a cluster-randomized trial | General Population | Indoor Residual Spraying (IRS) |
| 49 | Yang H, 2021 ​[49] | Myanmar | Efficacy and Safety of a Naphthoquine-Azithromycin Coformulation for Malaria Prophylaxis in Southeast Asia: A Phase 3, Double-blind, Randomized, Placebo-controlled Trial. | Age 5-18 years/ age 18-60 years | Perennial Malaria Chemoprevention |
| 50 | Ohnmar, 2012 ​[50] | Myanmar | Effects of malaria volunteer training on coverage and timeliness of diagnosis: a cluster randomized controlled trial in Myanmar. | General Population | Rapid Diagnostic Tests (RDT) & Artemisinin based Combination Therapy (ACT) |
| 51 | Phommasone, 2020 ​[51] | Myanmar | Mass drug administrations with dihydro artemisinin-piperaquine and single low dose primaquine to eliminate Plasmodium falciparum have only a transient impact on Plasmodium vivax: Findings from randomised controlled trials | General Population excluding pregnant women | Mass Drug Administration |
| 52 | Aung PL, 2019 [52]​ | Myanmar | Health education through mass media announcements by loudspeakers about malaria care: prevention and practice among people living in a malaria endemic area of northern Myanmar | Age 18-60 years | Information Education Communication (IEC) |
| 53 | Agius PA, 2020 ​[53] | Myanmar | Evaluation of the effectiveness of topical repellent distributed by village health volunteer networks against Plasmodium spp. infection in Myanmar: A stepped-wedge cluster randomised trial | General Population | Mosquito Repellent |
| 54 | Mclean, 2021 ​[54] | Myanmar | Mass drug administration for the acceleration of malaria elimination in a region of Myanmar with artemisinin-resistant falciparum malaria: a cluster-randomised trial | General Population excluding pregnant women | Mass Drug Administration |
| 55 | Saito M, 2021 [55] ​ | Myanmar | A randomized controlled trial of dihydro artemisinin-piperaquine, artesunate-mefloquine and extended artemether-lumefantrine treatments for malaria in pregnancy on the Thailand-Myanmar border. | Age 18-60 years | Artemisinin based Combination Therapy (ACT) |
| 56 | Ajayi IO, 2008 ​[56] | Nigeria | Assessment of a treatment guideline to improve home management of malaria in children in rural south-west Nigeria | General Population | Information Education Communication (IEC) |
| 57 | Onwujekwe, 2015 ​[57] | Nigeria | Effectiveness of Provider and Community Interventions to Improve Treatment of Uncomplicated Malaria in Nigeria: A Cluster Randomized Controlled Trial | General Population | Information Education Communication (IEC) |
| 58 | Balami AD, 2019 [58] ​ | Nigeria | Effects of a health educational intervention on malaria knowledge, motivation, and behavioural skills: a randomized controlled trial. | Antenatal Care Attendees | Information Education Communication (IEC) |
| 59 | Liu Jx, 2016 ​​[59] | Nigeria | Evaluation of SMS reminder messages for altering treatment adherence and health seeking perceptions among malaria care-seekers in Nigeria. | General Population | Rapid Diagnostic Tests |
| 60 | Meremikwu M, 2006 ​[60] | Nigeria | Artemether-lumefantrine versus artesunate plus amodiaquine for treating uncomplicated childhood malaria in Nigeria: randomized controlled trial | under 5 years of age | Artemisinin based Combination Therapy (ACT) |
| 61 | Omale UI, 2021 [61] ​ | Nigeria | Social group and health-care provider interventions to increase the demand for malaria rapid diagnostic tests among community members in Ebonyi state, Nigeria: a cluster-randomised controlled trial | General Population | Intervention to increase the demand of RDTs. |
| 62 | Onwujekwe O, 2015 ​[62] | Nigeria | Effectiveness of Provider and Community Interventions to Improve Treatment of Uncomplicated Malaria in Nigeria: A Cluster Randomized Controlled Trial | General Population | RDTs & ACTs. |

References:

[1] Li G, Yuan Y, Zheng S, Lu C, Li M, Tan R, et al. Artemisinin-piperaquine versus artemether-lumefantrine for treatment of uncomplicated Plasmodium falciparum malaria in Grande Comore island: an open-label, non-randomised controlled trial. Int J Antimicrob Agents 2022;60:106658. https://doi.org/10.1016/j.ijantimicag.2022.106658.

[2] Kofoed PE, Ursing J, Rodrigues A, Rombo L. Paracetamol versus placebo in treatment of non-severe malaria in children in Guinea-Bissau: A randomized controlled trial. Malar J 2011;10:1–8. https://doi.org/10.1186/1475-2875-10-148/TABLES/5.

[3] Pell C, Straus L, Phuanukoonnon S, Lupiwa S, Mueller I, Senn N, et al. Community response to intermittent preventive treatment of malaria in infants (IPTi) in Papua New Guinea. Malar J 2010;9:369. https://doi.org/10.1186/1475-2875-9-369.

[4] Sottas O, Guidi M, Thieffry B, Id MS, Dé Costerd L, Mueller I, et al. Adherence to intermittent preventive treatment for malaria in Papua New Guinean infants: A pharmacological study alongside the randomized controlled trial 2019. https://doi.org/10.1371/journal.pone.0210789.

[5] Mankadi PM, Jin Y. Effects of Door-to-Door Hang-Up Visits on the Use of Long-Lasting Insecticide-Treated Mosquito Nets in the Democratic Republic of the Congo: A Cluster Randomized Controlled Trial. Int J Environ Res Public Health 2021;18. https://doi.org/10.3390/IJERPH18179048.

[6] Atkinson JA, Bobogare A, Vallely A, Boaz L, Kelly G, Basifiri W, et al. A cluster randomized controlled cross-over bed net acceptability and preference trial in Solomon Islands: community participation in shaping policy for malaria elimination. Malar J 2009;8. https://doi.org/10.1186/1475-2875-8-298.

[7] Kere NK, Arabola A, Bakote’e B, Qalo O, Burkot TR, Webber RH, et al. Permethrin-impregnated bednets are more effective than DDT house-spraying to control malaria in Solomon Islands. Med Vet Entomol 1996;10:145–8. https://doi.org/10.1111/J.1365-2915.1996.TB00720.X.

[8] Leslie T, Rowland M, Mikhail A, Cundill B, Willey B, Alokozai A, et al. Use of malaria rapid diagnostic tests by community health workers in Afghanistan: cluster randomised trial. BMC Med 2017;15. https://doi.org/10.1186/S12916-017-0891-8.

[9] Leslie T, Mayan MI, Hasan MA, Safi MH, Klinkenberg E, Whitty CJM, et al. Sulfadoxine-Pyrimethamine, Chlorproguanil-Dapsone, or Chloroquine for the Treatment of Plasmodium vivax Malaria in Afghanistan and Pakistan: A Randomized Controlled Trial. JAMA 2007;297:2201–9. https://doi.org/10.1001/JAMA.297.20.2201.

[10] Soma DD, Zogo B, Hien DF de S, Hien AS, Kaboré DA, Kientega M, et al. Insecticide resistance status of malaria vectors Anopheles gambiae (s.l.) of southwest Burkina Faso and residual efficacy of indoor residual spraying with microencapsulated pirimiphos-methyl insecticide. Parasit Vectors 2021;14:58. https://doi.org/10.1186/S13071-020-04563-8.

[11] Kouyaté B, Somé F, Jahn A, Coulibaly B, Eriksen J, Sauerborn R, et al. Process and effects of a community intervention on malaria in rural Burkina Faso: randomized controlled trial. Malar J 2008;7:50. https://doi.org/10.1186/1475-2875-7-50.

[12] Tagbor H, Cairns M, Bojang K, Coulibaly SO, Kayentao K, Williams J, et al. A Non-Inferiority, Individually Randomized Trial of Intermittent Screening and Treatment versus Intermittent Preventive Treatment in the Control of Malaria in Pregnancy. PLoS One 2015;10. https://doi.org/10.1371/JOURNAL.PONE.0132247.

[13] Bassat Q, Mulenga M, Tinto H, Piola P, Borrmann S, Menéndez C, et al. Dihydroartemisinin-Piperaquine and Artemether-Lumefantrine for Treating Uncomplicated Malaria in African Children: A Randomised, Non-Inferiority Trial. PLoS One 2009;4. https://doi.org/10.1371/JOURNAL.PONE.0007871.

[14] Brogdon J, Dah C, Sié A, Bountogo M, Coulibaly B, Kouanda I, et al. Malaria positivity following a single oral dose of azithromycin among children in Burkina Faso: a randomized controlled trial. BMC Infect Dis 2022;22. https://doi.org/10.1186/S12879-022-07296-4.

[15] Louis VR, Bals J, Tiendrebéogo J, Bountogo M, Ramroth H, De Allegri M, et al. Long-term effects of malaria prevention with insecticide-treated mosquito nets on morbidity and mortality in African children: randomised controlled trial. Tropical Medicine & International Health 2012;17:733–41. https://doi.org/10.1111/J.1365-3156.2012.02990.X.

[16] Coulibaly B, Zoungrana A, Mockenhaupt FP, Schirmer RH, Klose C, Mansmann U, et al. Strong Gametocytocidal Effect of Methylene Blue-Based Combination Therapy against Falciparum Malaria: A Randomised Controlled Trial. PLoS One 2009;4. https://doi.org/10.1371/JOURNAL.PONE.0005318.

[17] Müller O, Traoré C, Kouyaté B, Yé Y, Frey C, Coulibaly B, et al. Effects of insecticide-treated bednets during early infancy in an African area of intense malaria transmission: a randomized controlled trial. Bull World Health Organ 2006;84:120. https://doi.org/10.2471/BLT.05.023150.

[18] Dambach P, Winkler V, Bärnighausen T, Traoré I, Ouedraogo S, Sié A, et al. Biological larviciding against malaria vector mosquitoes with Bacillus thuringiensis israelensis (Bti) – Long term observations and assessment of repeatability during an additional intervention year of a large-scale field trial in rural Burkina Faso. Glob Health Action 2020;13. https://doi.org/10.1080/16549716.2020.1829828.

[19] Sirima SB, Richert L, Chêne A, Konate AT, Campion C, Dechavanne S, et al. PRIMVAC vaccine adjuvanted with Alhydrogel or GLA-SE to prevent placental malaria: a first-in-human, randomised, double-blind, placebo-controlled study. Lancet Infect Dis 2020;20:585–97. https://doi.org/10.1016/S1473-3099(19)30739-X.

[20] Tiono AB, Ouédraogo A, Ouattara D, Bougouma EC, Coulibaly S, Diarra A, et al. Efficacy of Olyset Duo, a bednet containing pyriproxyfen and permethrin, versus a permethrin-only net against clinical malaria in an area with highly pyrethroid-resistant vectors in rural Burkina Faso: a cluster-randomised controlled trial. The Lancet 2018;392:569–80. https://doi.org/10.1016/S0140-6736(18)31711-2.

[21] Zeba AN, Sorgho H, Rouamba N, Zongo I, Rouamba J, Guiguemdé RT, et al. Major reduction of malaria morbidity with combined vitamin A and zinc supplementation in young children in Burkina Faso: a randomized double blind trial. Nutr J 2008;7:7. https://doi.org/10.1186/1475-2891-7-7.

[22] Dambach P, Baernighausen T, Traoré I, Ouedraogo S, Sié A, Sauerborn R, et al. Reduction of malaria vector mosquitoes in a large-scale intervention trial in rural Burkina Faso using Bti based larval source management. Malar J 2019;18:311. https://doi.org/10.1186/S12936-019-2951-3.

[23] Zongo I, Milligan P, Compaore YD, Some AF, Greenwood B, Tarning J, et al. Randomized Noninferiority Trial of Dihydroartemisinin-Piperaquine Compared with Sulfadoxine-Pyrimethamine plus Amodiaquine for Seasonal Malaria Chemoprevention in Burkina Faso. Antimicrob Agents Chemother 2015;59:4387. https://doi.org/10.1128/AAC.04923-14.

[24] Zoungrana A, Coulibaly B, Sié A, Walter-Sack I, Mockenhaupt FP, Kouyaté B, et al. Safety and Efficacy of Methylene Blue Combined with Artesunate or Amodiaquine for Uncomplicated Falciparum Malaria: A Randomized Controlled Trial from Burkina Faso. PLoS One 2008;3. https://doi.org/10.1371/JOURNAL.PONE.0001630.

[25] Coulibaly B, Sié A, Dah C, Bountogo M, Ouattara M, Compaoré A, et al. Effect of a single dose of oral azithromycin on malaria parasitaemia in children: a randomized controlled trial. Malar J 2021;20. https://doi.org/10.1186/S12936-021-03895-9.

[26] Chandramohan D, Zongo I, Sagara I, Cairns M, Yerbanga R-S, Diarra M, et al. Seasonal Malaria Vaccination with or without Seasonal Malaria Chemoprevention. New England Journal of Medicine 2021;385:1005–17. https://doi.org/10.1056/NEJMOA2026330/SUPPL_FILE/NEJMOA2026330_DATA-SHARING.PDF.

[27] Datoo MS, Natama MH, Somé A, Traoré O, Rouamba T, Bellamy D, et al. Efficacy of a low-dose candidate malaria vaccine, R21 in adjuvant Matrix-M, with seasonal administration to children in Burkina Faso: a randomised controlled trial. Lancet 2021;397:1809. https://doi.org/10.1016/S0140-6736(21)00943-0.

[28] Mbacham WF, Mangham-Jefferies L, Cundill B, Achonduh OA, Chandler CIR, Ambebila JN, et al. Basic or enhanced clinician training to improve adherence to malaria treatment guidelines: a cluster-randomised trial in two areas of Cameroon. Lancet Glob Health 2014;2:e346–58. https://doi.org/10.1016/S2214-109X(14)70201-3.

[29] Nsimba B, Guiyedi V, Mabika-Mamfoumbi M, Mourou-Mbina JR, Ngoungou E, Bouyou-Akotet M, et al. Sulphadoxine/pyrimethamine versus amodiaquine for treating uncomplicated childhood malaria in Gabon: A randomized trial to guide national policy. Malar J 2008;7:31. https://doi.org/10.1186/1475-2875-7-31.

[30] Grobusch MP, Lell B, Schwarz NG, Gabor J, Dörnemann J, Pötschke M, et al. Intermittent Preventive Treatment against Malaria in Infants in Gabon-A Randomized, Double-Blind, Placebo-Controlled Trial. J Infect Dis 2007;196:1595–602. https://doi.org/10.1086/522160.

[31] Oyakhirome S, Issifou S, Pongratz P, Barondi F, Ramharter M, Kun JF, et al. Randomized Controlled Trial of Fosmidomycin-Clindamycin versus Sulfadoxine-Pyrimethamine in the Treatment of Plasmodium falciparum Malaria. Antimicrob Agents Chemother 2007;51:1869. https://doi.org/10.1128/AAC.01448-06.

[32] Kremsner PG, Adegnika AA, Hounkpatin AB, Zinsou JF, Taylor TE, Chimalizeni Y, et al. Intramuscular Artesunate for Severe Malaria in African Children: A Multicenter Randomized Controlled Trial. PLoS Med 2016;13. https://doi.org/10.1371/JOURNAL.PMED.1001938.

[33] Alloueche A, Bailey W, Barton S, Bwika J, Chimpeni P, Falade CO, et al. Comparison of chlorproguanil-dapsone with sulfadoxine-pyrimethamine for the treatment of uncomplicated falciparum malaria in young African children: double-blind randomised controlled trial. The Lancet 2004;363:1843–8. https://doi.org/10.1016/S0140-6736(04)16350-2.

[34] Bélard S, Issifou S, Hounkpatin AB, Schaumburg F, Ngoa UA, Esen M, et al. A Randomized Controlled Phase Ib Trial of the Malaria Vaccine Candidate GMZ2 in African Children. PLoS One 2011;6. https://doi.org/10.1371/JOURNAL.PONE.0022525.

[35] Dicko A, Toure S, Traore M, Sagara I, Toure O, Sissoko M, et al. Increase in EPI vaccines coverage after implementation of intermittent preventive treatment of malaria in infant with Sulfadoxine -pyrimethamine in the district of Kolokani, Mali: Results from a cluster randomized control trial. BMC Public Health 2011;11:573. https://doi.org/10.1186/1471-2458-11-573.

[36] Dicko A, Brown JM, Diawara H, Baber I, Mahamar A, Soumare HM, et al. Primaquine to reduce transmission of Plasmodium falciparum malaria in Mali: a single-blind, dose-ranging, adaptive randomised phase 2 trial. Lancet Infect Dis 2016;16:674–84. https://doi.org/10.1016/S1473-3099(15)00479-X.

[37] Cairns ME, Sagara I, Zongo I, Kuepfer I, Thera I, Nikiema F, et al. Evaluation of seasonal malaria chemoprevention in two areas of intense seasonal malaria transmission: Secondary analysis of a household-randomised, placebo-controlled trial in Houndé District, Burkina Faso and Bougouni District, Mali. PLoS Med 2020;17. https://doi.org/10.1371/JOURNAL.PMED.1003214.

[38] Chandramohan D, Zongo I, Sagara I, Cairns M, Yerbanga R-S, Diarra M, et al. Seasonal Malaria Vaccination with or without Seasonal Malaria Chemoprevention. New England Journal of Medicine 2021;385:1005–17. https://doi.org/10.1056/NEJMOA2026330/SUPPL_FILE/NEJMOA2026330_DATA-SHARING.PDF.

[39] Maiga OM, Kayentao K, Traoré BT, Djimde A, Traoré B, Traoré M, et al. Superiority of 3 Over 2 Doses of Intermittent Preventive Treatment With Sulfadoxine-Pyrimethamine for the Prevention of Malaria During Pregnancy in Mali: A Randomized Controlled Trial. Clinical Infectious Diseases 2011;53:215–23. https://doi.org/10.1093/CID/CIR374.

[40] Maiga H, Opondo C, Chico RM, Cohee LM, Sagara I, Traore OB, et al. Overall and Gender-Specific Effects of Intermittent Preventive Treatment of Malaria with Artemisinin-Based Combination Therapies among Schoolchildren in Mali: A Three-Group Open Label Randomized Controlled Trial. Am J Trop Med Hyg 2022;107:796. https://doi.org/10.4269/AJTMH.21-1218.

[41] Rhee M, Sissoko M, Perry S, McFarland W, Parsonnet J, Doumbo O. Use of insecticide-treated nets (ITNs) following a malaria education intervention in Piron, Mali: a control trial with systematic allocation of households. Malar J 2005;4:35. https://doi.org/10.1186/1475-2875-4-35.

[42] Sissoko MS, Healy SA, Katile A, Zaidi I, Hu Z, Kamate B, et al. Safety and efficacy of a three-dose regimen of Plasmodium falciparum sporozoite vaccine in adults during an intense malaria transmission season in Mali: a randomised, controlled phase 1 trial. Lancet Infect Dis 2022;22:377–89. https://doi.org/10.1016/S1473-3099(21)00332-7.

[43] Sagara I, Ellis RD, Dicko A, Niambele MB, Kamate B, Guindo O, et al. A Randomized, Controlled, Phase 1 Study of the Safety and Immunogenicity of the AMA1-C1/Alhydrogel® + CPG 7909 Vaccine for Plasmodium falciparum Malaria, in Semi-immune Malian Adults. Vaccine 2009;27:7292. https://doi.org/10.1016/J.VACCINE.2009.10.087.

[44] Barry A, Issiaka D, Traore T, Mahamar A, Diarra B, Sagara I, et al. Optimal mode for delivery of seasonal malaria chemoprevention in Ouelessebougou, Mali: A cluster randomized trial. PLoS One 2018;13. https://doi.org/10.1371/JOURNAL.PONE.0193296.

[45] Bassat Q, Mulenga M, Tinto H, Piola P, Borrmann S, Menéndez C, et al. Dihydroartemisinin-Piperaquine and Artemether-Lumefantrine for Treating Uncomplicated Malaria in African Children: A Randomised, Non-Inferiority Trial. PLoS One 2009;4. https://doi.org/10.1371/JOURNAL.PONE.0007871.

[46] Aponte JJ, Aide P, Renom M, Mandomando I, Bassat Q, Sacarlal J, et al. Safety of the RTS,S/AS02D candidate malaria vaccine in infants living in a highly endemic area of Mozambique: a double blind randomised controlled phase I/IIb trial. The Lancet 2007;370:1543–51. https://doi.org/10.1016/S0140-6736(07)61542-6.

[47] Menéndez C, Bardají A, Sigauque B, Sanz S, Aponte JJ, Mabunda S, et al. Malaria Prevention with IPTp during Pregnancy Reduces Neonatal Mortality. PLoS One 2010;5. https://doi.org/10.1371/JOURNAL.PONE.0009438.

[48] Wagman JM, Varela K, Zulliger R, Saifodine A, Muthoni R, Magesa S, et al. Reduced exposure to malaria vectors following indoor residual spraying of pirimiphos-methyl in a high-burden district of rural Mozambique with high ownership of long-lasting insecticidal nets: entomological surveillance results from a cluster-randomized trial. Malar J 2021;20. https://doi.org/10.1186/S12936-021-03583-8.

[49] Yang H, Wang J, Liu H, Zhao Y, Lakshmi S, Li X, et al. Efficacy and Safety of a Naphthoquine-Azithromycin Coformulation for Malaria Prophylaxis in Southeast Asia: A Phase 3, Double-blind, Randomized, Placebo-controlled Trial. Clin Infect Dis 2021;73:e2470. https://doi.org/10.1093/CID/CIAA1018.

[50] Ohnmar, Tun-Min, San-Shwe, Than-Win, Chongsuvivatwong V. Effects of malaria volunteer training on coverage and timeliness of diagnosis: a cluster randomized controlled trial in Myanmar. Malar J 2012;11:309. https://doi.org/10.1186/1475-2875-11-309.

[51] Phommasone K, van Leth F, Peto TJ, Landier J, Nguyen TN, Tripura R, et al. Mass drug administrations with dihydroartemisinin-piperaquine and single low dose primaquine to eliminate Plasmodium falciparum have only a transient impact on Plasmodium vivax: Findings from randomised controlled trials. PLoS One 2020;15. https://doi.org/10.1371/JOURNAL.PONE.0228190.

[52] Aung PL, Pumpaibool T, Soe TN, Burgess J, Menezes LJ, Kyaw MP, et al. Health education through mass media announcements by loudspeakers about malaria care: prevention and practice among people living in a malaria endemic area of northern Myanmar. Malar J 2019;18:362. https://doi.org/10.1186/S12936-019-2985-6.

[53] Agius PA, Cutts JC, Oo WH, Thi A, O’Flaherty K, Aung KZ, et al. Evaluation of the effectiveness of topical repellent distributed by village health volunteer networks against Plasmodium spp. infection in Myanmar: A stepped-wedge cluster randomised trial. PLoS Med 2020;17. https://doi.org/10.1371/JOURNAL.PMED.1003177.

[54] McLean ARD, Indrasuta C, Khant ZS, Phyo AK, Maung SM, Heaton J, et al. Mass drug administration for the acceleration of malaria elimination in a region of Myanmar with artemisinin-resistant falciparum malaria: a cluster-randomised trial. Lancet Infect Dis 2021;21:1579. https://doi.org/10.1016/S1473-3099(20)30997-X.

[55] Saito M, Carrara VI, Gilder ME, Min AM, Tun NW, Pimanpanarak M, et al. A randomized controlled trial of dihydroartemisinin-piperaquine, artesunate-mefloquine and extended artemether-lumefantrine treatments for malaria in pregnancy on the Thailand-Myanmar border. BMC Med 2021;19. https://doi.org/10.1186/S12916-021-02002-8.

[56] Ajayi IO, Falade CO, Bamgboye EA, Oduola AMJ, Kale OO. Assessment of a treatment guideline to improve home management of malaria in children in rural south-west Nigeria. Malar J 2008;7:24. https://doi.org/10.1186/1475-2875-7-24.

[57] Onwujekwe O, Mangham-Jefferies L, Cundill B, Alexander N, Langham J, Ibe O, et al. Effectiveness of Provider and Community Interventions to Improve Treatment of Uncomplicated Malaria in Nigeria: A Cluster Randomized Controlled Trial. PLoS One 2015;10. https://doi.org/10.1371/JOURNAL.PONE.0133832.

[58] Balami AD, Said SM, Zulkefli NAM, Bachok N, Audu B. Effects of a health educational intervention on malaria knowledge, motivation, and behavioural skills: a randomized controlled trial. Malar J 2019;18. https://doi.org/10.1186/S12936-019-2676-3.

[59] Liu JX, Modrek S. Evaluation of SMS reminder messages for altering treatment adherence and health seeking perceptions among malaria care-seekers in Nigeria. Health Policy Plan 2016;31:1374. https://doi.org/10.1093/HEAPOL/CZW076.

[60] Meremikwu M, Alaribe A, Ejemot R, Oyo-Ita A, Ekenjoku J, Nwachukwu C, et al. Artemether-lumefantrine versus artesunate plus amodiaquine for treating uncomplicated childhood malaria in Nigeria: randomized controlled trial. Malar J 2006;5:43. https://doi.org/10.1186/1475-2875-5-43.

[61] Omale UI, Azuogu BN, Alo C, Madubueze UC, Oka OU, Okeke KC, et al. Social group and health-care provider interventions to increase the demand for malaria rapid diagnostic tests among community members in Ebonyi state, Nigeria: a cluster-randomised controlled trial. Lancet Glob Health 2021;9:e320–30. https://doi.org/10.1016/S2214-109X(20)30508-8.

[62] Onwujekwe O, Mangham-Jefferies L, Cundill B, Alexander N, Langham J, Ibe O, et al. Effectiveness of Provider and Community Interventions to Improve Treatment of Uncomplicated Malaria in Nigeria: A Cluster Randomized Controlled Trial. PLoS One 2015;10. https://doi.org/10.1371/JOURNAL.PONE.0133832.
